# Education Research: Sustained Implementation of Quality Improvement Practices Is Observed in Early Career Physicians Following a Neurology Resident QI Curriculum

**DOI:** 10.1212/NE9.0000000000200137

**Published:** 2024-06-10

**Authors:** Katherine Xiong, Rebecca K. Miller-Kuhlmann, Brian J. Scott, Zihuai He, Shefali Dujari, Carl Gold, Kathryn Kvam

**Affiliations:** From the Department of Neurology & Neurological Sciences (K.X., R.K.M.-K., B.J.S., Z.H., S.D., C.G., K.K.), and Quantitative Sciences Unit (Z.H.), Stanford School of Medicine, Stanford University, CA.

## Abstract

**Background and Objectives:**

The Accreditation Council for Graduate Medical Education and American Board of Psychiatry and Neurology expect engagement in quality improvement (QI) activities for all residents and practicing neurologists. Our neurology residency program instituted an experiential Neurology Residency QI Curriculum in 2015 for all residents. In this study, we aimed to characterize the role of QI engagement in the early-career paths of program graduates.

**Methods:**

We distributed an online survey evaluating QI training, scholarship, and leadership (before, during, and after residency training) to all individuals who graduated from our residency program (graduation years 2017–2021). Primary outcomes were QI project leadership or mentorship and QI scholarship (projects, posters, and publications) after residency. Predictors of these outcomes were also evaluated using Fisher exact test.

**Results:**

Twenty-nine of 50 graduates (58%) completed the survey. Median time from residency graduation was 3 years. Of the respondents, 14% actively participated in a QI project before residency, 83% during residency, and 48% after graduating. In addition, 41% had led or mentored a QI project and 34% had performed QI scholarship since residency. Fourteen percent of participants held formal roles in QI or patient safety, while 24% received formal full-time equivalents for QI work. Significant predictors (*p* < 0.05) of QI leadership included older age, time since graduation, rank, and participation in Clinical Effectiveness Leadership Training (CELT—an institutional QI faculty development course). Significant predictors (*p* < 0.05) of QI scholarship included older age, time since graduation, participation in CELT, and participation in QI scholarship during residency. QI training, participation, and/or project leadership before residency did not predict either QI leadership or scholarship after residency.

**Discussion:**

Many neurology residency graduates continued to lead QI projects and produce QI scholarship in the early years after graduation. However, receiving protected time for leadership and academic work in this area is uncommon. Our findings suggest that more infrastructure, including training, career development, and mentorship, can foster neurologists interested in leading in quality and patient safety. In academic models, promotion pathways that support academic advancement for faculty leading in QI are needed.

## Introduction

Engagement in quality improvement (QI) is required by the Accreditation Council for Graduate Medical Education (ACGME) for all medical residents, and application of improvement skills is part of the American Board of Psychiatric and Neurology Maintenance of Certification for practicing neurologists.^1,2^ However, how neurologists develop the skills necessary to engage in QI is not well defined, with few published neurology specialty-specific QI curricula.^3-6^

In a previous study, we found that a Neurology QI Curriculum (QIC) led to a significant increase in resident self-assessed confidence in most of the QI skills and a significant increase in QI project participation, from 39% participation in the precurriculum period to 77% (*p* = 0.023) in the postcurriculum period.^3^ However, few reports describe career outcomes following QI curricula in any specialty,^7-9^ and none describe early-career outcomes for neurology residency graduates.

To address this gap, our primary aim was to characterize the role that QI leadership and scholarship play in the careers of recent neurology residency graduates and identify predictors of early-career QI leadership and scholarship. Our secondary aim was to understand which aspects of our program's QIC were most relevant to early-career neurologists. We hypothesized that neurology residents involved in QI projects and scholarship during neurology residency would be more likely to be engaged in QI work in their early careers.

We believe our findings will be of interest to neurology training programs nationally, many of which are starting to adopt QI education programs, and to department leaders who may wish to recruit and support faculty interested in leadership and scholarship in QI.

## Methods

### Institutional Context

Our institution has implemented multiple programs to train faculty, trainees, and staff in QI skills and methodology. A Resident Safety Council (RSC) was established in 2014 with the intent to foster multidisciplinary, multidepartment, resident-led, QI projects. Since 2015, residents have had the opportunity to participate in the annual hospital-wide Quality Improvement & Patient Safety Symposium. The 5-month long Clinical Effectiveness Leadership Training course (CELT) was established in 2015 to promote clinician development and leadership in QI. It is primarily geared towards medical faculty and their interdisciplinary QI project team partners, but select residents and fellows can participate as part of a project team led by a faculty mentor and facilitated by a dedicated QI coach. Over the course of 10 protected, full-day sessions, participants receive didactics on QI skills and leadership from national topic experts, significant institutional project support, and dedicated mentorship. The Realizing Improvement through Team Empowerment program was developed in 2016 and available to all health care staff with the goal of promoting the development of sustainable, multidisciplinary improvement projects and QI knowledge through a 5-month long multidisciplinary training program.^10^

Our Neurology Residency Program is comprised of adult and child neurology trainees. The residency program implemented a formal residency QIC in 2015. Based on a needs assessment using semistructured interviews with residents and residency program and graduate medical education leadership, the goals of the QIC were to (1) promote reflective practice to identify gaps in care and (2) launch and support successful resident-led improvement initiatives.

Adult learning theories including cognitive apprenticeship and Kolb's experiential learning theory were applied in the initial curricular development of 2 separate QI experiential opportunities for the residency program and subsequent iterations.^11^ The first component is a monthly, resident-led, multidisciplinary Morbidity, Mortality, & Improvement (MMI) conference. Each resident is required to present at least once on a case and lead their peers through a fishbone analysis of the gaps in care to identify resident-led improvement ideas.

The second component is a 4-part workshop series within the residents' required weekly didactics, where resident-led project teams learn and apply A3 problem solving tools to a real project with a group faculty mentor. Workshops are led by a pair of QI-trained faculty members, with a total of ∼12 hours of didactic and team-based learning time. These began as a lecture-based format and transitioned to a flipped-classroom format in 2021 in response to resident feedback. Didactic content is focused on foundational knowledge and skills in defining current state, including process maps and run charts, conducting analysis using tools such as Pareto charts and 5 Whys, creating key driver diagrams, and developing a sustain plan.^3,12^ Project ideas are resident driven with many arising from MMI conference presentations. All residents are assigned to one of the project teams, but ongoing active participation in a QI project is not a program requirement. The graduating class of 2017 was the first to be offered at least 1 entire year of the curriculum.

### Study Design

All graduates of the Stanford Neurology Residency Program between 2017 and 2021 were eligible to participate in our study. These residents had participated in the Neurology QIC during their neurology training (postgraduate years 2–4) and had at least 1 year of clinical experience after residency at the time of the survey to ensure adequate time had passed to assess early-career impact of the curriculum.

### Assessment Tools

Survey design was informed by the study aim and review of the literature.^7,9,13^ A pilot online survey focused on QI experiences (prior, during, and after residency training) and their opinions of components of the Neurology QIC was developed and sent to 8 reviewers (7 faculty and 1 fellow) in June 2022. The survey was revised based on responses (n = 6). The final survey was distributed via Qualtrics and emailed to all 50 eligible residency graduates (eAppendix 1). Surveys were distributed from July to August 2022. All participants were eligible to receive a $20 gift card as an incentive upon completion of the survey.

Our primary outcomes were (1) QI project leadership or mentorship after residency and (2) QI scholarship after residency, where QI scholarship referred to any posters, abstracts or talks given at conferences or any publications on QI, value-based care, patient safety (PS), and/or QI education. In addition, survey items included demographic information, current practice characteristics, previous QI training, and experience as an active participant in a QI project before, during, and after residency to investigate possible predictors of early-career participation in QI activities. Active participation in a QI project was defined as “serving as a member of a team, with sustained involvement of a project through: problem and SMART goal development, key driver identification, intervention implementation, and data analysis.”

Secondary outcomes included assessment of self-reported confidence with QI work, self-reported impact of the curriculum on current practice, and the perceived usefulness of various components of the QIC using a 5-point Likert-style scale (for confidence and curricular impact: “strongly disagree,” “disagree,” “neutral,” “agree,” and “strongly agree”; for curricular component usefulness: “not at all useful,” “somewhat useful,” “moderately useful,” “very useful,” and “extremely useful”). Three open-ended response questions explored the QIC impact on participants' clinical practice, as well as the strengths and areas of opportunity for the curriculum.

### Data Analysis

In addition to the descriptive analysis of our primary outcomes, we performed an additional analysis on primary outcome predictors. Given the small sample size, we considered this analysis to be exploratory and descriptive, and correction for multiple comparisons was not performed. To assess the association between each predictor and “Led or mentored after residency” or “QI scholarship after residency,” we quantified the overall association using Fisher's exact test. For continuous variables, we calculated the odds ratios, 95% confidence intervals, and corresponding *p*-values per unit change in the predictors using univariate analysis with Firth's bias-reduced logistic regression. We present these results in Table 3.

Open-ended response question data were independently reviewed by 2 authors (K.X. and K.K.). A list of broad categories was developed as key topics emerged using the conventional content analysis approach.^14^

### Standard Protocol Approvals, Registrations, and Consents

Our institution determined this study to be exempt from institutional review, as the involvement of human subjects in the research activities was in a category (category 2) that was exempt from the regulation at 45 Code of Federal Regulations (CFR) 46 or 21 CFR 56. Each survey included a consent form with the study's description, benefits, risks, confidentiality statement, and contact information. Confidentiality was maintained through anonymous data collection.

### Data Availability

Anonymized data not provided in the article may be shared on request of qualified investigators for replication purposes.

## Results

### Participant Demographic and Practice Characteristics

Thirty of 50 eligible graduates (60%) responded to the survey, with 29 of the 30 respondents completing the survey (58% completion rate) including primary outcomes. Where data was missing for a given question, only the surveys with a valid response were included in the results. As a result, 29 surveys were used in the final analysis.

As summarized in Table 1, 72% were women and 59% were between 30 and 34 years old, with a median of 3 years since residency graduation at the time of survey completion. All respondents were actively completing or had completed a fellowship program. Of the respondents, 69% practiced in an academic setting, while the remainder were in community practices (24%) or combined academic-community practices (7%); and 34% were at the same institution where they completed residency. Of the 22 (76%) respondents in academic or academic-community practices, 14 were assistant professors, 4 were clinical instructors and 4 were fellows. Twenty-five participants had full time equivalent (FTE) distributions to disclose—the remaining 4 participants were completing fellowship. Of those reporting FTE breakdown, 6/25 (24%) had FTE allotted for QI work, ranging from 5% to 10%, with a median of 5%. Median FTE for clinical/patient care was 80% (range 14%–100%), 2% research (range 0%–86%), 0% education (range 0%–42.5%), and 0% other (0%–20%). Four graduates (14%) hold a formal leadership role in QI at their institution, ranging from specific program, divisional, departmental, and hospital levels.

**Table 1:** Demographics of Included Early Post-Residency Neurology Graduates, 2017–2021

	n = 29
Female, n (%)	21 (72)
Age, y, n (%)	
30–34	17 (59)
35–39	10 (34)
40–44	2 (7)
Median years since graduation	3
Fellowship training, n (%)	
Completed fellowship	29 (100)
Completed fellowship at Stanford	21 (72)
Current practice setting, n (%)	
Academic	20 (69)
Community	7 (24)
Other	2 (7)
Professional rank/title, n (%)	
Fellow	4 (14)
Instructor	4 (14)
Assistant professor	14 (48)
Nonacademic clinical practice	7 (24)
Current institution, n (%)	
Same as residency	10 (34)
Current FTE distribution, % median (range)	n = 25
Clinical/patient care	80 (14–100)
Education	0 (0–42.5)
Research	2 (0–86)
QI	0 (0–10)
Other	0 (0–20)
QI experience, n (%)	n = 29
Before neurology residency	
Had formal QI training	5 (17)
Actively participated in a project	4 (14)
Led a project	2 (7)
During residency	
Actively participated in a project	24 (83)
Led a project	15 (52)
QI scholarship	18 (62)
QI scholarship: Institutional	16 (55)
Poster or abstract	15 (52)
Invited talk or course	7 (24)
QI scholarship: Regional/national	11 (38)
Poster or abstract	7 (24)
Invited talk or course	3 (10)
Publication	9 (31)
Actively participated in institutional QI committee	4 (14)
After residency	
Actively participated in a project	14 (48)
Hold a formal role in QI-PS	4 (14)

Abbreviations: FTE = full-time equivalent; PS = patient safety; QI = quality improvement.

### QI Experience Before Residency

Before residency, 17% respondents had formal QI training, with 10% receiving training during medical school and 7% during medical or pediatric internship. Before residency, 14% participated in a QI project and just 2 (7%) led a QI project (Table 1).

### QI Experience During Residency

During residency, 83% of respondents actively participated in a QI project and 52% led a QI project. In addition, 62% participated in QI scholarship during residency, with 55% occurring at an institutional level and 38% occurring at a regional/national level (Table 1).

Three respondents had additional QI training (all participating in the CELT course) after residency during their fellowship.

### Primary Outcomes: QI Experience After Residency

Forty-one percent of respondents reported leading or mentoring a QI project after residency and 34% have performed QI scholarship (Table 2). QI scholarship occurred at both an institutional level (28%) and regional/national level (21%) in the form of abstracts/posters at conferences, publications in peer-reviewed journals, and presentations (Table 2).

**Table 2:** Primary Outcomes: Leadership and Scholarship in QI Among Early-Career Neurology Residency Graduates

	Yes, n (%); n = 29
Led or mentored a project after residency	12 (41)
No. of projects, median (range)	2 (1–4)
Participated in QI scholarship after residency	10 (34)
QI scholarship: Institutional	8 (28)
Poster or abstract	5 (17)
Invited talk or course	6 (21)
QI scholarship: Regional/national	6 (21)
Poster or abstract	6 (21)
Invited talk or course	1 (3)
Publication	4 (14)

Abbreviation: QI = quality improvement.

### Predictors of QI Leadership and/or Scholarship After Residency

Leadership after residency: In univariate analysis (Table 3), age (*p* = 0.03) and current clinical rank (*p* = 0.007) were significantly associated with QI project leadership after residency. A similar significant association was seen for number of years since graduation (*p* = 0.008). Participation in the CELT program during residency was also significantly associated with QI project leadership after residency (*p* = 0.021). However, other before residency experiences (including formal QI training, active project participation, and leading a project) and during residency experiences (including active project participation, leading a project, and participating in the Resident Safety Council) were not.

**Table 3:** Predictors of QI Leadership and Scholarship After Residency

	Led or mentored QI project, yes (n = 12), n (%)	Led or mentored QI project, no (n = 17), n (%)	Overall *p* value^a^	QI scholarship, yes (n = 10), n (%)	QI scholarship, no (n = 19), n (%)	Overall *p* value^a^
Women	9 (75)	12 (71)		8 (80)	13 (68)	
Men	3 (25)	5 (29)		2 (20)	6 (32)	
Age			0.03			0.021
<35	4 (33)	13 (76)		3 (30)	14 (74)	
≥35	8 (67)	4 (24)		7 (70)	5 (26)	
Years since graduation, mean	4.1	2.3	0.008^b^	3.9	2.6	0.04^b^
Fellowship institution			0.41			0.67
Same as residency	10 (83)	11 (65)		8 (80)	13 (68)	
Different than residency	2 (17)	6 (35)		2 (20)	6 (32)	
Current practice setting			1			1
Academic	8 (67)	12 (71)		7 (70)	13 (68)	
Community	3 (25)	4 (24)		2 (20)	5 (26)	
Other	1 (8)	1 (6)		1 (10)	1 (5)	
Professional rank/title			0.007			0.11
Fellow	0 (0)	4 (24)		0 (0)	4 (21)	
Instructor	0 (0)	4 (24)		1 (10)	3 (16)	
Assistant professor	10 (83)	4 (24)		8 (80)	6 (32)	
Nonacademic clinical practice	2 (17)	5 (29)		1 (10)	6 (32)	
Current institution			0.45			1
Different than residency	9 (75)	10 (59)		7 (70)	12 (63)	
Same as residency	3 (25)	7 (41)		3 (30)	7 (37)	
QI experience						
Before residency						
Had formal QI training	1 (8)	4 (24)	0.37	1 (10)	4 (21)	0.63
Participated in a project	1 (8)	4 (53)	0.37	1 (10)	4 (21)	0.63
Led a project	1 (8)	1 (6)	0.4	1 (10)	1 (5)	0.4
During residency						
Participated in a project	11 (92)	13 (77)	0.37	9 (90)	15 (79)	0.63
Led a project	8 (67)	7 (41)	0.42	7 (70)	8 (42)	0.39
Participated in QI scholarship	10 (83)	8 (47)	0.06	9 (90)	9 (47)	0.044
Participated in RSC	3 (25)	2 (12)	0.62	3 (30)	2 (11)	0.31
Participated in CELT	4 (33)	0 (0)	0.021	4 (40)	0 (0)	0.009
After residency						
Had formal QI training	2 (17)	1 (6)	0.55	1 (10)	2 (11)	1

Abbreviations: CELT = Clinical Effectiveness Leadership Training; QI = quality improvement; RSC = Resident Safety Council.

a Based on Fisher exact test that tests for the overall association except as noted.

b Based on bias-reduced logistic regression that tests for the association of each category relative to the reference group.

Scholarship after residency: Age (*p* = 0.021) and years since graduation (*p* = 0.04) were significantly associated with QI scholarship. Participation in the CELT program during residency was also significantly associated with scholarship after residency (*p* = 0.009). QI scholarship during residency was significantly associated with ongoing QI scholarship after residency (*p* = 0.044). However, other during residency experiences (active QI project participation, leading a project, participating in Resident Safety Council) and before residency experiences (formal QI training, active project participation, leading a project) did not predict QI scholarship after residency.

Respondents' sex, fellowship institution, current practice setting, current institution, and formal QI training after residency were not significantly associated with QI leadership or scholarship after residency.

### Additional Survey Findings

Forty-eight percent of respondents reported active participation in QI projects after residency, and 59% respondents agreed or strongly agreed with the statement that “[they] feel confident in their ability to successfully conduct QI work.” Fifty-nine percent also agreed or strongly agreed that “the Neurology QI Curriculum…significantly helped [their] career development,” with 1 respondent reporting they felt they were “a better clinician, especially in inpatient settings, because of [their] experience learning clinical neurology in an environment keenly focused on QI and analysis of medical errors, suboptimal trends, and care gaps/inequalities,” and another who described the curriculum as a “launching pad into the world of quality improvement.” Common themes across participant feedback on the impact of the QIC on their clinical practices included QI skill building, increased confidence in QI abilities, and heightened awareness for opportunities for improvement (Table 4). Even respondents who are not actively participating in QI work in their careers reported finding the curriculum helpful, with 1 respondent sharing that “while [they're] not doing QI work now, the curriculum helped [them] learn the potential academic and institutional possibilities regarding this type of work,” and another stating that they “do frequently think back on the curriculum trying to discern ways to improve [their] practice efficiency and improve care delivery to patients.”

**Table 4:** Strengths and Practice Impact of Neurology Resident QIC According to Study Participants

	Qualitative themes	Examples
Skills	QIC participants developed QI skills that are applicable to current practice	While I don't currently participate in a QI role, the process that was taught to me during residency helps me think about and solve problems that I encounter on a day-to-day basis in the clinic. The most important aspect is to understand the system and work within it to solve problems. Current practice and approach to QI systems-based issues has marked drivers from the residency curriculum, specifically the frameworks for approaching problem identification, key drivers, pareto conceptualization, and use of real time run charts.
Confidence	QIC participants have confidence in their QI skills	I am able to identify potential QI projects and I have a framework for investigation. I feel confident in developing SMART goals to measure success. Compared to my colleagues in fellowship, I have a much better understanding of the QI process. Although my career will not be focused on QI, I participate in departmental projects and provide input through committee participation.
Clinical	QIC contributed to sense of improvement in broader clinical abilities	I am a better clinician, especially in inpatient settings, because of my experience learning clinical neurology in an environment keenly focused on quality improvement and analysis of medical errors, suboptimal trends, and care gaps/inequities.
Projects	Real QI projects were helpful to develop skills	This is the kind of thing you really only learn by doing and practicing hands on. Having residents choose real, relevant projects was also helpful. Hands-on experience with leading a project was one of the highlights.
Awareness	Awareness of opportunities for improvement in current clinical settings	Makes me more conscious of areas within my organization that can be improved and gives some sense of how I might approach attempted change. While I'm not doing QI work now, the curriculum helped me learn the potential academic and institutional possibilities regarding this type of work, and it's nice to have that knowledge if the opportunity came up.
Time	Active engagement and participation in QIC or ongoing scholarship requires time (protected time)	Often unable to make lectures due to clinical obligations and participation in this often means taking even additional time from your days off, which is not feasible in an already busy training program. I think in general I am too busy with other aspects of my job to focus on formal QI work; that being said I do frequently think back on the curriculum trying to discern ways to improve my practice efficiency and improve care delivery to patients.

Abbreviations: QI = quality improvement; QIC = QI curriculum.

Our curricular needs assessment demonstrated that the reported percentage of respondents who rated a given curricular element as “very useful” to “extremely useful” (or 4–5 on the Likert Scale respectively) was 48% for A3 methodology, 45% for didactic lectures, 48% for resident-led teams with faculty members, 66% for the experience of working on resident identified QI projects, 72% for the curricular inclusion of real QI projects, and 76% for MMI conferences. The primary barrier to curricular engagement was time for participation, with multiple graduates reporting that they were “often unable to make lectures due to clinical obligations and participation,” and that having protected time for the curricular sessions would improve the curriculum. Respondents also shared that having more guidance in project scope and sustainability would have been helpful, noting that the frequency of “residents cycling through and graduating” makes iterations on projects challenging. Several respondents also shared that more guidance on publishing QI scholarship would have been helpful.

## Discussion

Our institution has previously implemented and described a multifaceted QIC for neurology residents.^3^ In this study, we describe the early-career outcomes of neurology residency graduates after exposure to a QIC. Our study demonstrates that active participation, leadership, and scholarship in QI was highest during residency, but nearly half of the respondents continued to participate in QI projects after residency, with over one-third continuing as project leaders/mentors and producing QI scholarship. Production of QI scholarship during residency was significantly associated with ongoing QI scholarship afterward, whereas higher clinical rank was associated with QI leadership, but not with QI scholarship. QI experience (training, participation, and leadership) before and during residency was not significantly associated with our primary outcomes, but participating in the Stanford CELT course—a 5-month long intensive QI leadership program—was associated with both QI leadership and scholarship. Age and years since graduation were also associated with both QI leadership and scholarship.

The ACGME currently requires that all residents receive training in QI processes,^15^ but there continues to be significant variability in training depending on the institution and department. Curricular structures can vary by target audience (whether specialty-specific vs general programs), duration (individual didactic lectures vs longitudinal experiences), modality (web-based modules, didactic lectures, project application), and curricular content.^16-19^ Several institutions, including ours, also have institutional multidisciplinary QI leadership development programs that can supplement resident education in QI. Unique to our QIC was the strong emphasis on the development and successful implementation of longitudinal QI projects in contrast to the predominantly didactics-based curricular structures that have been more widely adopted and published.^20-26^ To provide participants with an opportunity for real-time, hands-on application of QI tools learned in the accompanying didactics, most of these projects were conceived by residents, and all were led by residents with the support of faculty mentors with QI experience.

We believe a unique strength of our study is a focus on application of curricular material beyond the training period and correlation with career outcomes, achieving program evaluation at Kirkpatrick level 3 (level 3: application of learning to the workplace).^27^ Specifically, our study's analysis of rates of QI engagement as project leaders and mentors is hypothesized to reflect the ongoing application of curricular learning to participants' clinical practice while QI scholarship is felt to serve as a proxy for results affecting system quality and patient outcomes. In our literature review, most studies focus on curricular outcomes at a Kirkpatrick level 1–2 (level 1: reaction/satisfaction, level 2: learning/knowledge)—knowledge of QI methodologies, domains, and confidence in QI skills as well as curricular satisfaction.^16-18^ A handful of studies have looked at the curricular impact on patient outcomes.^28-31^ Others have reported participation in QI projects and QI scholarly activity.^32,33^ However, most of these studies measure engagement and scholarship during the active curricular period (or immediately after). Our study found that 62% reported QI scholarship during residency. These rates appear consistent with other reported rates in the literature for QI scholarship in residents receiving program-wide QIC, with one study reporting 70% (7/10) of their resident teams presenting posters at professional meetings after implementation of a QIC for internal medicine residents.^32^ A survey of pediatric residency program directors found that 38%–92% residents had submitted a QI abstract to a professional meeting, with higher submission rates in programs with more faculty involved in QI.^33^ Somewhat higher rates of resident scholarship have been described in resident research programs, including in neurology,^34^ although notably these often have a curricular requirement for scholarship.^35^

Rates of ongoing QI engagement among our graduates were lower than during residency but still moderate. The only studies on career outcomes after QIC exposure we could find were of dedicated QI fellowship or training tracks. In addition to lectures, these programs provided mentorship with institutional QI leaders, required QI project completion, and offered participants protected time (either elective time during residency or protected funded time for QI work—typically 75%–80% time for QI work if after residency).^7,9,36-39^ Such programs are designed to support individuals interested in acquiring knowledge, pursuing scholarship, and/or aspiring toward careers in health care quality. One study of a 2-year health care leadership track in quality within residency found that 87% of graduates (1–7 years later) were still engaged in QI, with 45% having leadership roles, 40% had presented at a national conference and 30% had published in a peer-reviewed journal. Fifty-five percent had financial support for QI, most through salary (62%) but some through grants (19%), bonus/stipend (12%), or industry (8%). Graduates who were at least 3 years out were significantly more likely to have financial support for QI.^7^ Another study of graduates of the Veterans Affairs (VA) Chief Resident in Quality and Safety (CRQS) program 1–9 years after graduation found that 70% were still conducting QI projects, 35% were conducting research related to QI/PS, and 31% held formal QI or PS leadership positions.^9^ Other studies have examined outcomes after postgraduate QI/PS fellowship programs for clinicians seeking advanced health care quality skills and leadership training and have found high impact of the curriculum and high rates of subsequent leadership roles.^36,37^

While we found less ongoing QI engagement in our study compared with these studies, this is not unexpected given the differences in program participants and structure. Our curriculum was administered to all residents rather than a self-selecting cohort with expressed interest in QI. These other curricula were generally longer, more intensive, and tended to be cross-departmental. Such structural differences may offer specific benefits and challenges to a curriculum. For example, cross-departmental QI fellowships and training tracks oftentimes are structured in a manner that offers more protected time for QI education and scholarship, which is desirable for project success.^16,40^ In addition, such programs can allow for consolidation of resources, including faculty expertise; however, such cross-departmental programs can require increased coordination and agreement on curricular components.^16,17,41^ Specialty-specific programs may offer opportunities for more customized education, access to within-department faculty mentors who can provide both project and career support, and flexibility to work within the scheduling constraints of the training program.

Of interest, neither QI experience (training, project participation and/or leadership) before residency or additional training after residency predicted QI leadership or scholarship after residency. This suggests the high impact that in-residency training and leadership opportunities have on subsequent early-career success. We found that participation in our institution's intensive project-based CELT program during residency was a significant predictor of both QI leadership and scholarship. The structure of the CELT program is similar to other institutions' QI fellowships or tracks, with participants across specialties applying to engage in a 5-month longitudinal course focused on developing leadership and QI skills. Its association as a predictor of postgraduate leadership and scholarship may be in part because of the declared interest of participants in the program. Of interest, participation in CELT after residency did not have the same predictive effect on QI leadership or scholarship, although this finding might be limited by the fact that median time since residency graduation for study participants was 3 years. Our study also found age (*p* = 0.03), years since graduation (*p* = 0.008), and current clinical rank (*p* = 0.007) to be predictors of QI leadership. This may suggest that residency graduates early in their careers may not have (or may not be allocated) as much time for nonclinical duties. A previous study found that the reported percentage of time dedicated to QI work increased with increasing experience across alumni from the VA CRQS program, with alumni with 1 year of postgraduate experience dedicating 20% of their time compared with 34% reported by alumni with 5 or more years; however, this difference was not statistically significant.^9^

Many of our graduates were continuing to produce scholarship in QI, half of which were at the regional/national level including abstracts, talks, and/or publications. This suggests that several of our graduates were achieving the milestones needed for promotion with QI as an area of focus. A recent study evaluating the impact of the integration of a QI portfolio in the University of California San Francisco Department of Medicine's promotion process found that 55% of the QI portfolio submissions came from individuals at the assistant professor rank, compared with 28% from the associate professor and 16% from the full professor rank, which may suggest that QI is a burgeoning career venue, particularly for early-career clinicians.^42^ However, achieving a better understanding of the need for these career development pathways to a successful career is limited by the dearth of published research in this area. Understanding the depth and breadth of QI scholarship by neurologists is limited by definitions (quality, health services research, implementation science, educational studies) and the breadth of journals in which these studies are published.

Notably, it is important to acknowledge that publications may not capture the full extent of QI scholarship being produced. While this may be a result of the typically local and/or institutional nature of QI work, it may reflect the comparatively limited opportunities for QI scholarship distribution and dissemination. In our study, we found that participation in QI scholarship during residency was significantly associated with continued QI scholarship after residency training. This suggests that mentorship in producing scholarship in QI was an essential step in independently leading and producing scholarship after residency.

While we found that many graduates were continuing to actively participate in QI work, a much smaller proportion had dedicated FTE for QI and the median FTE was 5%. Only half of those with current QI leadership roles receive FTE for their positions. This suggests that there is a gap in infrastructure for supporting leaders in QI despite increasing institutional and national emphasis on quality, safety, and value. Some systems have more organized infrastructure to support training, leadership, and career advancement in this area. Of interest, in the VA CRQS alumni study, those graduates not employed by the VA were more likely than VA employees to report organizational barriers to QI, including lack of recognition or incentive related to career advancement, lack of mentorship or faculty development, lack of team members with suitable knowledge, and challenges accessing/collecting data.^9^ The role QI leadership and scholarship plays in professional advancement is a very recently established one, with development of the first QI portfolio defining standards for academic promotion for internal medicine physicians occurring only in 2009^43^ and the publication of the first interprofessional health care improvement portfolio occurring recently in 2022.^44^ This, in conjunction with the previously mentioned challenges associated with dissemination of QI scholarship, demonstrate the clear need for ongoing development and formalization of QI career pathways and advancement.

Limitations of this work include the lack of a control group and small sample size, with possibility for a nonresponse bias (40% of the eligible participants did not respond). While we found a correlation between our QIC and early-career scholarship and leadership, we cannot prove causation. It is possible that other factors including institutional culture, increased attention to quality and PS at the Graduate Medical Education level, and expanding opportunities for QI scholarship and career development nationally also contributed. We also acknowledge potential limitations to generalizability because our residency uniquely offers exposure to the QIC across all 3 years of neurology training, which may contribute to the proportion of ongoing QI scholarship and leadership observed in our graduates. In addition, all respondents completed a fellowship after residency, which is not universal at other institutions. Most of our respondents practice in an academic setting, which is higher than the current national rate of 33% of neurologists in academia, based on American Academy of Neurology Insights 2022 audience profile data,^45^ and may limit the generalizability of our findings to broader national practice patterns.^46^ That being said, several graduates in community and hybrid practice settings reported ongoing QI engagement. Given the small sample size, predictor analysis was considered exploratory and descriptive, and as a result, the associations are marginal. Adjustment for potential confounders should be considered in future larger scale studies.

Our study offers a unique insight into the early-career trajectories of our neurology residency program's graduates. It highlights QI as an avenue of ongoing scholarship and leadership for many of our early-career graduates. For most of our graduates, first exposure to QI methodology and experiences began during the neurology residency QIC. The structure of our curriculum, with integration of real QI projects and MMI case conferences, was well received. However, dedicated time for these projects is a barrier to more meaningful participation. As neurology programs continue to develop QI curricula, including project-based opportunities for the application of QI methodology, protected time for project participation, and further education on producing QI scholarship and QI/PS career opportunities may be valuable skills to integrate.

Collectively, our findings suggest that more infrastructure is needed to support neurologists interested in leading in quality and PS. Training, career development, and mentorship are needed. In academic models, promotion pathways that support academic advancement for faculty who are successfully achieving regional and national recognition are essential.

## Appendix Authors

**Table:**

Name	Location	Contribution
Katherine Xiong, MD	Department of Neurology & Neurological Sciences, Stanford University, CA	Drafting/revision of the manuscript for content, including medical writing for content; major role in the acquisition of data; study concept or design; analysis or interpretation of data
Rebecca K. Miller-Kuhlmann, MD	Department of Neurology & Neurological Sciences, Stanford University, CA	Drafting/revision of the manuscript for content, including medical writing for content; study concept or design; analysis or interpretation of data
Brian J. Scott, MD	Department of Neurology & Neurological Sciences, Stanford University, CA	Drafting/revision of the manuscript for content, including medical writing for content; study concept or design; analysis or interpretation of data
Zihuai He, PhD	Department of Neurology & Neurological Sciences, and Quantitative Sciences Unit, Stanford School of Medicine, Stanford University, CA	Drafting/revision of the manuscript for content, including medical writing for content; study concept or design; analysis or interpretation of data
Shefali Dujari, MD	Department of Neurology & Neurological Sciences, Stanford University, CA	Drafting/revision of the manuscript for content, including medical writing for content; study concept or design; analysis or interpretation of data
Carl Gold, MD, MS	Department of Neurology & Neurological Sciences, Stanford University, CA	Drafting/revision of the manuscript for content, including medical writing for content; study concept or design; analysis or interpretation of data
Kathryn Kvam, MD	Department of Neurology & Neurological Sciences, Stanford University, CA	Drafting/revision of the manuscript for content, including medical writing for content; major role in the acquisition of data; study concept or design; analysis or interpretation of data

## Glossary

ACGME: Accreditation Council for Graduate Medical Education
CELT: Clinical Effectiveness Leadership Training
CFR: Code of Federal Regulations
CRQS: Chief Resident in Quality & Safety
FTE: full time equivalent
MMI: Morbidity, Mortality, & Improvement
PS: patient safety
QI: quality improvement
QIC: QI curriculum
RSC: resident safety council
VA: Veterans Affairs
